# A balancing act: Primary care midwives screening for fetal growth restriction- a focus group study

**DOI:** 10.1016/j.ijnsa.2025.100439

**Published:** 2025-10-23

**Authors:** Mariëlle van Roekel, Dominique Kramer, Ank de Jonge, Arie Franx, Jens Henrichs, Corine J. Verhoeven

**Affiliations:** a Amsterdam UMC location Vrije Universiteit Amsterdam, Midwifery Science, Amsterdam, the Netherlands; b Midwifery Academy Amsterdam Groningen, Inholland, Amsterdam, the Netherlands; c Amsterdam Reproduction and Development Research Institute, Amsterdam the Netherlands; d University of Groningen, University Medical Center Groningen, Department of Primary and Long-term Care, Groningen, the Netherlands; e Department of Obstetrics and Gynaecology, Erasmus University Medical Center, Rotterdam, the Netherlands; f Amsterdam Public Health Research Institute, Quality of Care, Amsterdam, the Netherlands; g Division of Midwifery, School of Health Sciences, University of Nottingham, Nottingham, United Kingdom; h Department of Obstetrics and Gynaecology, Maxima Medical Centre, Veldhoven, the Netherlands

**Keywords:** Fetal growth restriction, Screening, Decision making, Interprofessional relationships, Midwifery

## Abstract

**Background:**

Fetal growth restriction in low-risk pregnancies often remains undetected, despite its association with perinatal morbidity and mortality. While technical improvements in screening have been widely studied, little is known about how autonomous midwives in primary care settings navigate screening for fetal growth restriction in daily practice.

**Aim:**

To better understand how midwives in low-risk settings navigate the screening process for fetal growth restriction, and to identify opportunities for improvement.

**Methods:**

An interpretative qualitative study was conducted based on five online focus groups with 21 participants, including midwives and senior midwifery students across the Netherlands. Reflexive thematic analysis was carried out by a multidisciplinary team.

**Findings:**

Midwives described screening for fetal growth restriction as a balancing act shaped by diagnostic uncertainty, ambiguous guidelines, interprofessional mistrust and moral responsibility. Five themes were identified: managing screening uncertainty, adapting to women’s care needs, coping with a lack of trust in collaboration with secondary care, balancing tradition and innovation within professional identity, and maintaining professional confidence.

**Conclusion:**

Screening for fetal growth restriction is not only a technical act but also a relational and value-laden practice. Midwives’ practices reflect a continuous negotiation between societal reliance on technology, the limitations of current tools and guidelines, interprofessional tensions, and the relational and moral responsibilities of care. Improving screening therefore requires more than technical solutions: it calls for clear guidelines, respectful collaboration, and supportive structures that enable midwives to practice with confidence and trust.

**What is already known?**

• Fetal growth restriction often goes undetected in women without traditional risk factors, making detection in low-risk pregnancies particularly important.
• Current screening methods in low-risk pregnancies have limited sensitivity and uncertain impact on perinatal outcomes.
• Research has largely focused on improving diagnostic accuracy, with limited attention to the experiences of midwives who perform the screening in practice.

Alt-text: Unlabelled box

**What this paper adds?**

• This study shows how midwives navigate tensions in screening for fetal growth restriction by balancing diagnostic uncertainty, systemic ambiguity, interprofessional mistrust, women’s care needs and societal expectations.
• It demonstrates that screening for fetal growth restriction is not only a technical act but also a relational and value-laden practice.
• It identifies priorities for practice and policy, including co-designed protocols, stronger interprofessional trust, and support for addressing the emotional and moral dimensions of care.

Alt-text: Unlabelled box

## 1 Introduction

Fetal growth restriction is a complex condition in which the fetus experiences malnutrition and often also chronic or even acute hypoxia (Malhotra et al., 2019). In most cases it results from placental insufficiency which hinders the fetus’ ability to reach its growth potential and thereby increases the risk of perinatal morbidity, mortality, and long-term mental and somatic disorders (Barker, 2006). While there is no cure, intensified surveillance including cardiotocography and doppler assessment along with timely delivery, may prevent stillbirth and perinatal asphyxia (Melamed et al., 2021). Early detection of fetal growth restriction is therefore considered essential in lowering these risks (VZinfo.nl, 2024).

Although etiologically identical, fetal small-for-gestational-age is used as proxy in screening for fetal growth restriction (Melamed et al., 2021). Relph et al. (2023) found that most antenatally misdiagnosed small-for-gestational-age infants were born to low-risk women lacking common screening risk factors such as hypertensive disorders or smoking. This is concerning, as antenatally unidentified small-for-gestational-age carries a four- to five-fold increased risk of stillbirth compared to those antenatally identified (Gardosi et al., 2013).

Detecting growth restriction in low-risk populations, remains a persistent challenge (Melamed et al., 2021). In most countries, fundal height assessment with selective ultrasonography based on medical risk factors is the standard screening method (Khalil et al., 2024; Morris et al., 2024). However, with a sensitivity of approximately 25 % (13 %−30 %), the majority of small-for-gestational-age fetuses remain undetected.(Bais et al., 2004; Vieira et al., 2022) Some countries, such as France, routinely perform third-trimester ultrasonography achieving higher sensitivity rates around 40 % (31 %−46 %) (Ego et al., 2023). However, evidence that this approach reduces perinatal mortality is lacking (Al-Hafez et al., 2020; Henrichs et al., 2019).

Approximately 70 % of small-for-gestational-age fetuses are constitutionally small but healthy, raising concerns about overtreatment (Gordijn et al., 2016). Conversely, growth restriction can occur in fetuses classified as appropriate-for-gestational-age, risking undertreatment when fetal smallness is the only indicator (Gordijn et al., 2016). To address this, strategies focusing on fetal growth trajectories, such as growth velocity and crossing centiles, have recently been introduced to better differentiate between constitutionally small-for-gestational-age and growth restriction, as well as to improve detection in fetuses above the 10th percentile (Kamphof et al., 2023; van Roekel et al., 2023). However, their effectiveness in achieving these goals requires further investigation. Moreover, ultrasound estimates of fetal weight can misestimate newborn weight by approximately 10 % (Lindström et al., 2023).

Given the limited effectiveness of current screening methods, research has primarily focused on enhancing technical accuracy. Yet screening is not merely a diagnostic act; it also entails interpretation, interaction, and context-sensitive decision-making. Improving the detection of fetal growth restriction in low-risk populations offers particular potential for better outcomes, yet the mechanisms underlying this process remain underexplored. In countries where primary care midwives work autonomously, they provide continuity to care to low-risk women and play a central role in this process. They assess fetal growth, inform women, and decide when referral is needed. However, little is known about how midwives navigate the uncertainties involved in this screening process. The aim of this study is therefore to better understand how primary care midwives navigate screening for fetal growth restriction in low-risk pregnancies.

## 2 Methods

### 2.1 Design

To address this aim, we employed an interpretative qualitative design using focus groups. Focus groups are a suitable data collection method to facilitate dynamic group interaction, allowing participants to discuss both diverse and shared perspectives on screening for fetal growth restriction (Keeley et al., 2016). An interpretive approach was used to gain a deeper understanding of midwives' experiences with the screening process ().

### 2.2 Setting

The study was conducted in the Netherlands, where primary care midwives are autonomous healthcare professionals providing antenatal, intrapartum, and postpartum care to low-risk women. If risk factors or complications arise, women are referred to obstetrician-led care and may return to midwife-led care if diagnostic findings are normal (Midwives, 2014).

### 2.3 Participants and recruitment

Participants included practicing midwives and student midwives who had completed most of their internships. During these internships, students perform fetal growth assessments under the supervision of qualified midwives. Including students allowed us to capture how future professionals in midwifery begin to engage with screening for fetal growth restriction.

Our aim was to have five focus groups of four to six participants, the first of which served as a pilot (Hennink et al., 2019). Purposive sampling was used to achieve heterogeneity through variation in age, experience, practice location, practice setting, practice size, third trimester ultrasound screening strategy and population characteristics. Inclusion criteria were: midwifery students in the final phase of training or registered midwives being actively involved in antenatal care. There were no formal exclusion criteria beyond availability for participation. Although students followed the same bachelor of midwifery program, variation in internship placements provided diverse experiences.

Midwives were recruited through a flyer that was posted on a social media group for midwives, and a website connecting researchers, pregnant women, and maternity care professionals and teachers, www.childbirthnetwork.nl. The flyer provided a brief study description. Interested midwives subsequently received a detailed study-information leaflet via e-mail.

### 2.4 Ethics

Our study was reviewed and approved by the Medical Ethics Committee of Amsterdam University Medical Centers (reference number 2022.0866). The study complied with the ethical principles of the Declaration of Helsinki, and measures were taken to ensure confidentiality, voluntary participation, and the right to withdraw at any time. All participants provided written informed consent.

### 2.5 Data collection

The topic guide was developed using Ménage’s (2016) framework, which highlights midwifery-specific evidence-based decision-making across four sources: the midwife, resources, research, and the woman. It featured open-ended questions with prompts in these areas. Midwives were asked to discuss their experiences with screening for fetal growth restriction (the midwife), their perspectives on current guidelines (research), the screening strategies they used (resources), and how pregnant women influenced decision-making (the woman). As the discussions progressed, issues related to the current working environment, such as values, professional culture, and policies, emerged naturally, with the interviewer actively encouraging midwives to delve deeper into these topics.

Five online focus groups were conducted by the first (MvR) and second (DK) authors in March and April 2024. The initial session served as a pilot to test the topic guide. Adjustments were made, based on participant feedback and critical reflection, primarily refining the topic order to improve discussion flow. Minor revisions in question order and wording followed the second focus group. The final topic guide is provided in supplement S1.

Following the pilot with student midwives, four additional focus groups were conducted: three with midwives and one with student midwives. Each session was audio recorded, and member checks were performed to enhance accuracy and credibility. The focus group discussions lasted between 88 and 115 min.

### 2.6 Analyses

Discussions were transcribed using Microsoft Teams and the audio recordings were manually anonymised and reviewed to correct any inaccuracies. We employed Braun and Clarke's (2019) reflexive thematic analysis, to identify and interpret patterns of meaning. The analytical process involved familiarisation with the data, generation of initial codes, and iterative development and refinement of themes through constant comparison and team discussions. Adopting Anderson’s (2019) interpretive stance, we examined how midwives make sense of screening for fetal growth restriction in daily practice, considering clinical, emotional, and organisational contexts (Anderson et al., 2019). Reflexivity was maintained through critical reflection on our own professional backgrounds and collective dialogue, and diverging interpretations were valued as analytically enriching. For trustworthiness, we followed Lincoln and Guba (1985) criteria; credibility was supported through member checks and reflexive engagement; transferability through thick description of the setting, participants and data; dependability through an audit trail; and confirmability through multidisciplinary analysis, reflexive practice, and verbatim quotations. Coding and theme development were supported by MAXQDA 2022 (version 22.1.1), which facilitated data analyses while interpretation remained a researcher-driven process. The final code tree is provided in Supplement S2.

### 2.7 Researchers’ characteristics and reflexivity

MvR and DK conducted the focus groups and data analysis. MvR, a PhD student and midwife with experience in both community and hospital settings, and a background in social psychology, contributed an in-depth understanding of the challenges of screening for fetal growth restriction. DK, who was trained in Health Sciences, brought a public health and interdisciplinary perspective to complement the clinical and psychosocial lenses. CV, a hospital-based midwife and qualitative researcher, provided guidance throughout. The inclusion of AF (obstetrician and researcher), AdJ (primary care midwife and midwifery researcher), and JH (developmental psychologist and epidemiologist) further enhanced reflexivity by incorporating diverse disciplinary viewpoints. The research team included people of different genders, which contributed to a diversity of perspectives.

## 3 Findings

### 3.1 Participant characteristics

In total, 24 midwives initially registered for the focus groups; however, three withdrew— two for personal reasons and one due to work-related commitments— resulting in 21 midwives across five focus groups. For practical reasons, the student midwives were placed in the same group. Midwives came from different regions in the Netherlands, varied in age (21–70), and had varying working experience levels (<5 to >30 years). The sample included senior students and practising midwives working in urban, rural, group, locum, and caseload settings (Table 1).

**Table 1:** Characteristics of the participants.

	Participant	Practice region^a^	Setting	Age (range)^b^	Experience (years)^b^	Position
Group 1	1	North	Urban/Town	21–30	–	Senior Student
	2	North	Urban/Town	21–30	–	Senior Student
	3	North/Middle	Urban/Town	21–30	–	Senior Student
	4	North/South	Urban/Town	21–30	–	Senior Student
Group 2	5	North	Urban/Town	21–30	–	Senior Student
	6	North/Middle	Town	31–40	–	Senior Student
	7	Middle	Urban/Town	21–30	–	Senior Student
	8	South	Urban	41–50	–	Senior Student
	9	Middle/South	Rural/Town/Urban	21–30	–	Senior Student
Group 3	10	South	Town	21–30	< 5	Locum midwife
	11	North	Urban	41–50	11- 15	Midwife in group practice
	12	South	Rural	31–40	11 - 15	Caseload^c^
	13	South	Urban	51–60	26 - 30	Midwife in group practice
Group 4	14	Middle	Town	41–50	21 - 25	Midwife in group practice
	15	North	Urban	31–40	10 - 15	Midwife in group practice
	16	North	Town	21–30	6 - 10	Midwife in group practice
	17	North	Urban	21–30	< 5	Locum midwife^d^
Group 5	18	North	Urban	61–70	16 - 20	Midwife in group practice
	19	South	Town	31–40	6 - 10	Midwife in group practice
	20	Middle	Urban	21–30	< 5	Locum midwife
	21	Middle	Urban	21–30	< 5	Locum midwife

a Practice regions in the Netherlands correspond to the provinces used for holiday scheduling (‘vakantiespreiding’).

b Experience grouped in 10 year (age) or 5 year (experience) range for privacy.

c Caseload midwifes provide one-on-one care, acting as the sole provider for each client.

d Locum midwives are self-employed professional who work across multiple midwifery practices.

### 3.2 Synthesis and interpretation

The following themes represent our interpretative analysis of how midwives navigate screening for fetal growth restriction in low-risk pregnancies. These five themes reflect the evidence-based, relational, systemic, societal, and personal dimensions of practice, and illustrate how midwives manage tensions and responsibilities in screening. Five key themes were identified: (1) Managing screening and detection uncertainty, (2) Adapting to women’s care needs, (3) Coping with a lack of trust in collaboration with secondary care, (4) Balancing tradition and innovation within professional identity and (5) Maintaining professional confidence.

#### 3.2.1 Managing screening and detection uncertainty

This theme illustrates the challenges that midwives encounter in screening for fetal growth restriction. It also reflects how midwives navigate uncertainty in the absence of conclusive tools or standardized protocols, balancing clinical reasoning with guideline constraints and personal judgment.

Midwives recognized the detection of fetal growth restriction as a core professional responsibility and demonstrated a strong desire to improve detection,. However, they also struggled with the suboptimal reliability of both abdominal palpation and biometry ultrasound as well as with the ambiguity of screening and referral protocols.

*"It’s the core of our profession, isn’t it? Detecting hypertension and monitoring growth.....those are the main reasons why people come to us. Of course, I’m also here for the social aspect, but that’s [detecting growth restriction] the core of the medical side. And well, identifying hypertension is going well, but fetal growth, yes, sorry but honestly, we're doing a terrible job with that, really terrible. We’re getting it wrong with our hands and we’re getting it wrong with the ultrasound…"* (Participant 13).

Despite recognising these limitations, many midwives personally expressed reasonable trust in their own palpation techniques, feeling confident in detecting most growth abnormalities. In contrast, newly qualified midwives and students, citing limited experience, relied more on ultrasounds, valuing their perceived objectivity.

Regarding palpation, midwives demonstrated considerable variation in their preferred strategy. Most relied on anatomical landmarks, i.e. fundal height estimates in relation to the pubic symphysis, umbilicus, or xiphoid process. Others preferred to measure fundal-symphysis height using a tape-measure, citing greater accuracy. A few midwives adopted both strategies to mitigate uncertainty. Interestingly, some midwives felt that tape-measurement introduced greater interobserver variability, leading them to question its added value.

*"The anatomical reference points can vary significantly and don’t clearly match either of the two options, you know? But with centimetres, I feel more confident saying, ‘Yes, this is it.’"* (Participant 16)

*"We used to do that [tape measurements] for a long time, but we found that the variability between us was too large, so we started questioning its added value."* (Participant 14)

Midwives noted that limiting third-trimester fundal height palpations to two caregivers could reduce interobserver variability, but this was rarely implemented in group practices due to logistical constraints and client preferences, illustrating the tension between recommendations and practical realities.

Uncertainty extended to the interpretation of screening results and their implications for care. Conflicting views on the benefits and harms of various approaches, particularly concerns about false positives causing client stress, led to variation in practices. While some adhered strictly to the Dutch national guidelines offering selective screening, others adopted routine third-trimester biometry or low-threshold biometry without specific concern for abnormal growth.

"*Um, well, basically we don't perform biometry ultrasounds; we do offer position ultrasounds, and if these are performed before 36 weeks, they also include biometrics. But we don’t usually perform standard growth ultrasounds."* (Participant 10)

Conversely, some midwives deviated from the guidelines in the opposite direction, choosing not to perform indicated ultrasounds for cases such as high body mass index when fundal height measurements seemed reassuring. Despite diverse screening practices, midwives agreed that ultrasound alone should not be the sole basis for induction of labour, emphasizing limited reliability and that small-for-gestational-age does not equal growth restriction.

Midwives appreciated the clarity of certain aspects of the national guidelines, such as referral thresholds, but also highlighted some gaps. They called for clearer indications regarding biometry in low-risk pregnancies and more specific protocols for screening and managing slow growth patterns. Opinions varied on how to interpret slow growth patterns. Most felt that referrals were sometimes excessive, e.g. when a baby’s percentile dropped by 20–30 percentiles but other indicators, such as maternal perception of movement, were reassuring. Others noted that babies who were large-for-gestational-age at 20 weeks were particularly at risk of ending up small-for-gestational-age, underscoring the importance of follow-up in cases of slowing growth. These gaps in guidance often led to care decisions being perceived as arbitrary, particularly in ambiguous situations. One participant pointed to inconsistent use of early biometry setpoints across hospitals, revealing how institutional differences led to confusion:

“*In [HOSPITAL], we don’t include the structural anomaly scan in relation to biometric measurements for abnormal growth. In [HOSPITAL], we should include it.”* (Participant 14)

Midwives attributed inconsistencies to discrepancies between national guidelines and regional or hospital-specific protocols, which they felt hindered the provision of consistent care. While some expressed frustration with how these inconsistencies impacted clinical decision-making, others valued the autonomy and flexibility that ambiguity provided.

#### 3.2.2 Adapting to women’s care needs

This theme captures how midwives navigate the relational aspects of care during the screening process, balancing attentiveness to women’s emotional needs with concerns about inadvertently increasing anxiety, given the limitations of existing screening tools.

All midwives expressed a strong commitment to tailoring their care to meet women’s individual needs. Reassuring clients about their baby’s health was consistently identified as a central aspect of midwifery care. Many midwives linked frequent requests for ultrasounds to underlying anxiety, noting that these requests often stemmed from a desire for measurable reassurance that everything was well. Aware of this need, midwives generally believed that addressing this anxiety through meaningful conversations was more effective in fostering client confidence than simply complying with ultrasound requests. However, they also acknowledged that successfully reducing anxiety through dialogue alone was one of the most challenging aspects of their care.

Midwives differed in how they approached these requests. Some voiced concern that fulfilling such requests might unintentionally amplify anxiety, while others justified facilitating an ultrasound by emphasizing the woman’s piece of mind, downplaying potential harm.

*“ If she really needs those ultrasounds and it helps her feel better, then sure, go for it. But I believe that just talking to her and reassuring her is the real way to help."* (Participant 6)

Nevertheless, many midwives admitted feeling powerless when conversations alone failed to alleviate concerns, often resulting in complying with the requests despite initial reservations.

Simultaneously, midwives emphasized the importance of validating women’s concerns and recognizing them as experts on their own pregnancies. Even in the absence of clinical indications of abnormal growth, midwives felt it was important to acknowledge the potential value of maternal intuition.

*“I always think, maybe this lady senses something that I don’t feel or see, and I really try to go along with it.”* (Participant 11)

This illustrates a tension they struggled with: wanting to take such concerns seriously, while also adhering to the principle that ultrasounds should only be performed when clinically indicated. Midwives observed that the nature and frequency of ultrasound requests varied considerably across different client populations. Those working in urban areas with a high proportion of expats noted that these clients were often accustomed to more frequent ultrasounds and actively requested them. In contrast, midwives working in rural areas, smaller practices, or serving predominantly migrant populations reported that clients were generally more reluctant to undergo ultrasounds or could be reassured through conversations, resulting in fewer requests for third-trimester scans.

*““I think I have clients who prefer not to have an ultrasound; they’d rather endure things, even when it’s medically advised.”* (Participant 12)

Midwives emphasized the importance of educating clients about the limitations of ultrasound and normalizing a broad range of findings to reduce stress and build confidence. They stressed the need for clear and empathetic communication, which they felt was often lacking in referrals to external sonography facilities or hospitals, leading to increased maternal stress or confusion. In contrast, midwives conducting ultrasounds in their own practices felt that this setting allowed for more personalized and thoughtful communication, providing greater reassurance to clients.

*“Well, I’ve noticed in my practice…. and that’s because we do all the ultrasounds in-house, that it’s a huge advantage, because I think I’m more cautious in my communication with women than the average obstetric resident*.” (Participant 19)

#### 3.2.3 Coping with a lack of trust in collaboration with secondary care

This theme reveals how midwives navigate collaboration with secondary care. Their reflections reveal how perceived imbalances in trust and care philosophy shape their approach to screening for fetal growth restriction and referral.

While effective collaboration between primary and secondary care is essential for providing safe and comprehensive maternity care, many midwives reported challenges and a lack of trust in these partnerships. This significantly impacted their approach to managing suspected growth restriction referral and follow-up.

A key issue contributing to mistrust in the referral process is the perception that clients referred to secondary care for suspected small-for-gestational-age are not always referred back to primary care when parameters normalize or increased fetal surveillance is no longer required. Midwives partly attributed this to secondary care providers' reluctance to refer clients back to primary care. They perceived that secondary care providers sometimes exploit ambiguities in the guidelines, which clearly define referral thresholds but vaguely describe back-referral criteria, leaving room for inconsistent practices.

“*Somehow I always feel that the hospitals in our region are sending them in the direction of oh yes, you’re in the right place now, we have good medical care here, so just stay here*.” (Participant 21)

Midwives believed that their approach, characterized by personal attention and continuity, better suited women’s needs than the more protocolized and intervention-focused secondary care. They often felt that women received less personal attention in secondary care. This led to dismay about the loss of important aspects of clients’ autonomy when women were transferred. Several midwives found this morally challenging, as it conflicted with their values of relational care and respect for women’s autonomy.

*“But that’s the case nowadays: the paradox between primary and secondary care is that people who are fine, who have no issues, receive very personalized care— we know you, and you can always call, and we always answer. But when the shit hits the fan, you’re in a place where no one knows who’s on duty next. That’s the strange thing about our system.”* (Participant 15)

To cope, some midwives disengaged, feeling powerless to influence the situation despite caring for their clients. Others actively directed women to the hospital with which they had better working relationships, proactively following up with referred clients to assess the need for continued secondary care, adjusting their own referral thresholds (e.g., lowering the referral cut-off from <p10 to <p5), or incorporating Doppler assessments unilaterally to avoid unnecessary referrals.

*“There is a big difference between these two hospitals, so if clients have not made a choice yet, we kind of steer them towards which hospital to go to.”* (Participant 12)

While these strategies provided a sense of control, they also caused frustration, underscoring their desire for better collaboration with secondary care providers.

However, midwives also acknowledged that some clients preferred to remain in secondary care, perceiving it as a safer option, even though midwives viewed this as “a false sense of security.” This preference left some midwives feeling powerless, wondering why others did not recognize the quality and value of midwifery care.

*"Yeah, because they get ultrasounds more often [in secondary care], you know? Everything is measured and visualised. In primary care, it sometimes feels like people think, ‘Oh… they’re just feeling my belly, is that ít?’ And that feels a bit unfair.”* (Participant 1)

*“We work with a lot of expats who would prefer secondary care, uhm, but well, it's not always, I think, a better place, uhm, so well, that often frustrates us.”* (Participant 15)

A recurring issue was the criticism midwives received from secondary caregivers, particularly paediatricians, when small-for-gestational-age cases were missed. They expressed frustration at being blamed in hindsight for not performing ultrasounds, even when there was no clear indication to do so. Some noted that performing ultrasound often deflected criticism, regardless of its clinical relevance. While midwives recognized the importance of professional feedback to improve care, they called for balanced expectations and compassionate professional relationships.

Another issue influencing midwives’ lack of trust in secondary care was the disparity in ultrasound quality standards. While primary care midwives have to meet specific qualifications to perform ultrasound, they complained that in secondary care, ultrasound was sometimes performed by residents without proper training and qualifications, raising concerns about the quality of care.

*“I think it’ s really terrible that, as primary care midwives, we really have to prove our quality and hand in our logbook regularly and then she is seen by a resident who doesn’t even have an ultrasound certificate and the care process is determined based on that ultrasound.”* (Participant 8)

Midwives who reported positive collaborations with secondary care attributed this to mutual trust, respect, and clear agreements. They noted that women were discharged back to primary care when secondary surveillance was no longer needed. Some midwives described incorporating umbilical artery Doppler assessments in collaboration with secondary care, highlighting the benefits of regular contact for resolving doubts and fostering mutual learning. They also emphasized the need for midwives as a profession to take a more proactive role in improving internal collaboration and strengthening partnerships with secondary care.

*I’m not dissatisfied with how things are going with referrals, in general we do get a lot referred back […] I feel that my team and I are taken very seriously when it comes to referrals.”* (Participant 16)

#### 3.2.4 Balancing tradition and innovation within professional identity

This theme reflects how midwives construct their professional identity in response to evolving technological norms. It reveals a shared struggle to reconcile traditional midwifery values with external expectations for control and standardization. Midwives described navigating these pressures in daily practice striving to maintain their a hands-on, client-focused approach while responding to what they perceived as growing societal and client-driven demand for technology-based care. While midwifery traditionally values physiological care and minimal intervention, the increasing reliance on ultrasound has created friction. Many midwives considered intuition — developed through experience — as a defining skill that distinguishes them from residents, who often adhere to protocols more rigidly. They emphasized the role of gut feeling in detecting growth restriction, particularly when ultrasound findings appear normal.

Concerns about societal pressures to manage risk and control inherently uncertain outcomes were widespread. Older midwives emphasized the challenge of gaining the trust of today’s generation of women, who tend to place greater value on technological interventions to control these risks. Midwives viewed this shift as problematic, not because they opposed technology, but because clients were often unaware of the potential interventions that unnecessary ultrasound screening could trigger.

The tension between tradition and technology was also evident within the midwifery profession. Across all groups, midwives advocated preserving traditional approaches, emphasizing hands-on assessment and confidence in their skills. While recognizing the value of ultrasound, many supported selective screening and questioned whether performing ultrasound undermines palpation skills, leading to unnecessary biometry. They were also concerned that over-reliance on ultrasound could increase interventions.

*“We don’t perform them [third trimester ultrasound] routinely and I’m really happy we don’t, because I feel that otherwise we would really be over-medicalizing.”* (Participant 19)

Some acknowledged that integration of technology is inevitable and described criticism they had encountered for adopting a less restrictive approach to third-trimester ultrasonography. These experiences revealed different perspectives within the profession.

*“We live in a society that seeks a lot of control, right? 'Measuring is knowing' has become the standard in everything [.………] but when you conduct an ultrasound, there’s a lot of discussion within the midwifery community about whether it’s necessary.”* (Participant 14)

Others described how incorporating umbilical artery Doppler into their practice or shared care facility, enhanced their ability to promote physiological care and maintain continuity by better differentiating between small-for-gestational-age and growth restriction. They saw this as a way to support physiological care and continuity by avoiding unnecessary intervention.

*“But the cases between the P5 and P10 with a good Doppler, for us those are just follow-up cases in our care. Recently, I’ve had fetuses where we knew they would be small, but the PI measurements were good every week, so we just awaited spontaneous onset [of labour], and the parents really appreciated that.”* (Participant 18)

#### 3.2.5 Maintaining professional confidence

This theme sheds light on the emotional and moral burden involved in making screening decisions. It shows how all midwives, especially those early in their careers, navigate self-doubt, fear of error and the desire to provide responsible physiological care.

Midwives described how screening for fetal growth restriction placed emotional weight on their clinical decisions, often confronting them with the limits of certainty. Students in particular struggled to balance the fear of missing critical signs with the expectation of avoiding unnecessary referrals. This responsibility often led to moral distress and self-doubt. The prospect of being held accountable, through perinatal audit or litigation, intensified these concerns, especially among less experienced midwives. Students felt particularly vulnerable, after witnessing adverse perinatal outcomes during internships in obstetric-led settings, which made the risk feel more immediate than in midwifery-led placements.

*“My fear of a stillbirth or that I haven’t referred enough, but at the same time, I don’t want to refer too much because I want to safeguard the physiology. So yeah, that’s where I’m stuck in between.”* (Participant 7)

In contrast, experienced midwives described a gradual shift in how they related to uncertainty. Rather than eliminating fear or doubt altogether, they spoke of learning to live with it, developing trust in their own judgement and a more grounded acceptance of the inherent unpredictability of midwifery care. Over time, this capacity to tolerate ambiguity became part of what defined their professional confidence, even though their fear of overlooking complications never fully disappeared.

*“I’ve come to accept that everyone can make mistakes at some point; it is what it is.”* (Participant 11)

Past difficult cases often had a dual influence on care: they made midwives more cautious, sometimes hindering their ability to remain fully objective in similar situations, while simultaneously strengthening their confidence in personal decision-making by reinforcing their reliance on experience and intuition.

Developing and maintaining professional confidence was seen as essential. Midwives emphasised that learning to live with imperfection was part of becoming resilient in the face of clinical ambiguity. While experienced midwives spoke of gradually accepting uncertainty as intrinsic to their role, students described how coming to terms with the inevitability of mistakes remained one of the most challenging aspect of their learning.

## 4 Discussion

In this study we aimed to better understand how autonomous midwives navigate screening for fetal growth restriction in low-risk pregnancies and in the findings we identify five interwoven themes; managing screening uncertainty, adapting to women’s care needs, coping with a lack of trust in collaboration with secondary care, balancing tradition and innovation, and maintaining professional confidence. Together these themes reflect tensions between evidence-based care, relational responsibilities, systemic constraints, societal expectations, and personal-professional values. Our analysis further suggests that ambiguous guidelines, fragmented collaboration, and a professional climate marked by retrospective judgement and auditing further complicated midwives’ work, fuelling uncertainty about when and how to act. This uncertainty contributed to variation in screening practices and influenced how midwives weighed clinical, relational, and institutional demands.

Our findings demonstrate that midwives considered detecting growth restriction as a core responsibility, yet they felt uncertainty about their ability to meet this task because of the limited reliability of both palpation and ultrasound in low-risk pregnancies. This reflects a deeper challenge: how to make responsible decisions when tools are fallible, guidance unclear, and outcomes unpredictable. Although routine ultrasound increases the detection of small-for-gestational-age and is cost-neutral compared to selective screening, it also leads to higher induction rates without consistent improvement in perinatal outcomes (Al-Hafez et al., 2020; Caradeux et al., 2019; Henrichs et al., 2022), illustrating the unresolved tension between timely detection and potential overtreatment.

Our analysis also reveals that ambiguity in national guidelines compounded these challenges. Midwives described how vague or inconsistent protocols, particularly regarding the management of slowing growth, left significant room for interpretation. Consistent with Marijnen et al. (2022) midwives reported regional variation in how screening protocols were applied, often because of local agreements or hospital preferences. In the absence of clear guidance or a clearly superior strategy, screening practices diverged. While some midwives adhered strictly to selective ultrasound protocols, others performed low-threshold or even routine third-trimester scans. This variation reflects broader uncertainty about best practice and aligns with a recent systematic review showing that defensive behaviours are particularly common in clinical ‘grey zones’, contexts where evidence is inconclusive and professional views diverge (Lorenc et al., 2024).

The findings of the current study illustrate how midwives adapted to women’s care needs by balancing attentiveness to emotional concerns with their own hesitations about increasing anxiety or overusing ultrasound. This reveals the relational dimension of the screening process. Uncertainty in this context shaped midwives’ responses to requests for non-indicated ultrasounds, which women often value for reassurance or a sense of control (de Jong-Pleij et al., 2013; Skelton et al., 2024; Westerneng et al., 2019). Midwives expressed a desire to respect these needs but also voiced concern about overuse. Echoing earlier research (Favaretto and Rost, 2024; Thomas et al., 2017), they questioned the clinical value of reassurance scans and worried that growing reliance on ultrasound may erode midwifery skills and shift expectations towards more medicalised care. Despite these concerns, most midwives admitted that they often complied with women’s requests, particularly when anxiety persisted after counselling. This pattern where perceived patient pressure contributes to increased testing and referrals, is also observed in other healthcare contexts (Little et al., 2004). Such dynamics illustrate that midwives must balance evidence-based restraint with relational responsiveness, even when guidelines offer little clarity. While some reflected critically on this concession, others saw it primarily as a pragmatic response to client expectations. Those who were more liberal in offering ultrasounds sometimes perceived implicit disapproval from colleagues, suggesting that internal group norms may also contribute to professional pressure (Romijn et al., 2018; Tajfel and Turner, 1986). Together these dynamics illustrate that screening decisions are shaped not only by clinical factors, but also by moral reasoning, professional identity and social expectations, underscoring that screening for fetal growth restriction is not merely a technical act but a relational and value-laden practice.

The analyses also indicate that midwives experienced challenges in coping with a lack of trust in secondary care, reflecting unequal and mistrustful relationships with obstetric colleagues. This was shaped by ambiguous guidelines and perceived imbalances in referral and back-referral practices. Ambiguity in the guidelines was experienced as a barrier to consistent referrals and as a source of interprofessional mistrust. Previous work found that midwives often felt overruled by obstetricians, reinforcing these dynamics (Beier et al., 2024; Romijn et al., 2018; Schulz and Wirtz, 2025). Our analysis extends this by illustrating how diagnostic uncertainty and ambiguous guidelines interact, as reflected in perceived practice variation and interprofessional collaboration challenges.

We identified key elements essential to interprofessional trust, including clear communication, consistent back-referral, and shared protocols. When these elements were present, midwives described collaborative relationships marked by mutual respect and joint decision-making. Prior research also suggests that co-created guidelines and explicit referral criteria can foster consistency and strengthen interprofessional trust (Adeyemo et al., 2022). Building on this evidence, our analysis underscores the need for guidelines that are both explicit and value-based, allowing room for clinical judgement, client preferences, and responsible resource use (Porter, 2009).

We found that perceived hindsight judgement was a key source of emotional strain, with midwives feeling unfairly held accountable for missed small-for-gestational-age cases despite the diagnostic limitations. Similarly, Foster et al. (2023) found that judgement and blame can undermine midwives’ autonomy, especially when hindsight judgments ignore the complexity of real-time care. Weltens et al. (2019) add that disciplinary procedures and audits may reinforce overly cautious or defensive practices. This resonates with the concept of moral distress, where professionals know the right course of action but are unable to act due to institutional or system-related constraints (Foster et al., 2022; Jameton, 1984; Rost et al., 2025). In our study, evaluative pressure within diagnostic uncertainty appeared to contribute to moral and emotional burden, helping to explain the defensive practices some midwives described.

Midwives also voiced that they were confronted with balancing tradition and innovation, suggesting that midwives negotiated their professional identity between traditional hands-on midwifery values and growing societal and professional expectations for technology-based care. This struggle resonates with Sinatti et al. (2025), who described how midwives shoulder the emotional burden of reconciling relational and technocratic care logics, especially in times of disagreement or uncertainty. Rather than functioning as opposites, these models often coexist in practice, with midwives absorbing the tensions they generate. By conceptualising this work as emotional labour, comparable to 'watchful attendance' during birth, Sinatti et al. (2025), offer a valuable lens for understanding screening for growth restriction as a similarly emotionally charged task. It requires midwives to navigate clinical ambiguity, relational responsibility, and moral accountability, often resulting in moral strain as they must weigh competing values such as safety versus autonomy, or reassurance versus overuse, without clear ethical guidance. This may help explain why midwives in our study struggled to justify either action or inaction when clinical uncertainty allows for interpretation while perceived retrospective judgement remains high.

Our results also revealed that the participating midwives were engaged in maintaining professional confidence. This finding also highlights the emotional and ethical challenges midwives face in screening decisions, including fear of making errors. Managing growth restriction screening confidently does not necessarily stem from eliminating uncertainty, but rather from learning to live with it. This professional resilience seemed to grow with experience, suggesting that professional confidence develops overtime. Yet, such resilience is not a given. Parry et al. (2024) showed that many midwives consider leaving the profession due to emotional strain and a lack of support. A shift towards a younger, less experienced workforce may increase the risk of defensive practices in response to uncertainty, perceived scrutiny, or institutional pressure. We found support for this concern, suggesting that without adequate institutional and collegial support, the ability to integrate uncertainty into confident practice may be compromised. Therefore, mentoring, supervision, and open dialogue may help evolving confidence and reduce uncertainties. Kool et al. (2023) similarly emphasise the value of supportive workplace cultures in helping new midwives navigate the realities of complex care.

Midwives pointed to technical areas for improvement, particularly regarding ultrasound quality and communication practices. In the Netherlands, performance reviews are required in primary care, but not always enforced in secondary care (BEN, 2025). Midwives advocated for minimum volume norms to ensure ultrasound quality with sonographer competence and sufficient experience identified as key conditions. This recommendation is supported by existing research, although the optimal threshold remains unclear (Verfaille et al., 2020). They also stressed the importance of empathetic communication during scans, which has been shown to reduce maternal stress and shape perceptions of care (Pulliainen et al., 2019). Improving relational dynamics with secondary care was seen as a way to foster more aligned decision-making, and help mitigate defensive practices that arise when professionals operate in silos and feel under scrutiny. Future research should explore how the organisation of ultrasound services, guidelines, and relational or political dynamics shape ultrasound quality and referral decisions, and whether these factors constrain or support midwives in navigating the balancing act between relational, moral and, evidence-based dimensions of screening for fetal growth restriction.

### 4.1 Strengths and limitations

One strength of this study included the inclusion of diverse participant group, representing a broad spectrum of midwives across the Netherlands. Moreover, by fostering an atmosphere of openness and reflection, midwives were supported to share a wide range of experiences, facilitating in-depth discussions. The use of multiple focus groups, separate sessions for students and professionals, and multidisciplinary analysis contributed to the credibility and richness of the findings. Trustworthiness was enhanced by applying Lincoln and Guba (1985) criteria.

However, some limitations should be noted. Although separate student groups created a safer environment for sharing, they may have limited intergenerational dialogue. The online format improved accessibility but occasionally affected the natural flow of discussion. As with all focus group research, the presence of peers and facilitators may have influenced midwives' responses, including potentially introducing social desirability bias. On the other hand, the discussion with other colleagues may have helped midwives to articulate their own ideas and feelings clearly.

## 5 Conclusion

Our study provides insight into how midwives working in primary care settings navigate screening for fetal growth restriction amid systemic ambiguity, interprofessional tensions, and moral responsibility. Their practices reflect a continuous negotiation between societal reliance on technology and the limitations of current tools, guidelines, and interprofessional collaboration, showing that screening is not only a technical act, but also a relational and value laden practice. While striving to detect growth restriction and prevent adverse outcomes, midwives also aim to preserve the core values of their professional identity: personalised and relational care. Improving screening therefore requires more than technical refinements: it calls for clear, co-designed protocols, support for midwives’ professional development, and space to reflect on its moral and emotional dimensions. Ultimately, progress depends on creating conditions that enable midwives to negotiate this balancing act in their daily care and live with uncertainty as part of confident practice.

## Funding information

The work for the current study was funded within the research programme Doctoral Grant for teachers with project number 023.018.034, financed by the Netherlands Organization for Scientific Research. The funding organisations had no role in study design, data collection, data analysis, data interpretation, writing of the scientific article, or the decision to submit the paper for publication.

## Author agreement

We agree the article is the author’s original work and has not received prior publication or is under consideration for publication elsewhere. All authors have seen and approved the manuscript being submitted.

## CRediT authorship contribution statement

**Mariëlle van Roekel:** Writing – original draft, Visualization, Validation, Project administration, Methodology, Investigation, Formal analysis, Data curation, Conceptualization. **Dominique Kramer:** Writing – original draft, Project administration, Investigation, Data curation. **Ank de Jonge:** Writing – review & editing, Supervision, Methodology, Conceptualization. **Arie Franx:** Writing – review & editing, Formal analysis. **Jens Henrichs:** Writing – review & editing, Formal analysis. **Corine J. Verhoeven:** Writing – review & editing, Supervision, Methodology, Formal analysis, Conceptualization.

## Declaration of competing interest

We have no conflict of interest to declare.

## Appendix Supplementary materials

Image, application 1

Image, application 2
