# Supplementary material for: A balancing act: Primary care midwives screening for fetal growth restriction- a focus group study
Source: Int J Nurs Stud Adv. 2025 Oct 23;9:100439. doi: 10.1016/j.ijnsa.2025.100439 (PMC12607044; doi:10.1016/j.ijnsa.2025.100439)
Supplement: Supplementary file 1 [file mmc1.docx]

**Supplement 1. Final topic guide**

**English version**

| Time | Topics | Question | Sub-topics |
| --- | --- | --- | --- |
| 10’ | Opening question: Assessment the current situation | How do you experience the current protocols/guidelines within your perinatal network (VSV) and the national guideline regarding FGR screening and management?  How would you rate the current policies on a scale from 1 to 10? | (With post its):  • What are considerations for you not to rate lower and higher? Write on post its. • Discuss post its |
| 20’ | Exploring experiences  and decision-making considerations | How do you experience detecting FGR as a healthcare provider?  What challenges do you face in detecting FGR in clinical practice?  Have there been experiences that were particularly meaningful to you in FGR detection? |  |
| 30’ | Current care provision | Screening  What techniques / tools do you use for screening for FGR and what are your considerations and experiences?  Referral  What are considerations and experiences when referring to secondary care?  Uncertainty  How do you manage uncertainty in decision-making? | Screening   - Screening reliability - Palpation vs. ultrasound - Dopplers - Interpreting and managing slowing growth   Referral   - Factors influencing decision-making - Experiences and dilemmas - Variations in referral criteria between hospitals |
| 15’ | Influence of the pregnant client | How does the pregnant woman influence the (medical) decision-making process? | - What is effective (or ineffective) in reassuring clients? - How do client expectations for ultrasounds influence your decision-making? |
| 15’ | Opportunities for future improvements | What needs, preferences, or improvements do you see for the future? | (Getting back at post its if necessary)   - How can the previously given rating be improved? |
| 05’ | Closing question | Is there anything else you would like to add on this topic? |  |
