# Supplementary material for: A balancing act: Primary care midwives screening for fetal growth restriction- a focus group study
Source: Int J Nurs Stud Adv. 2025 Oct 23;9:100439. doi: 10.1016/j.ijnsa.2025.100439 (PMC12607044; doi:10.1016/j.ijnsa.2025.100439)
Supplement: Supplementary file 2 [file mmc2.docx]

**Balancing tradition and innovation within professional identity**

Positioning amid diverging views on a shifting professional identity

Trusting the gut feeling

The woman needs to be taken seriously

Struggling with interobserver reliability

Grief for women’s loss of personal care in action oriented secondary care

Mutual trust ensures good collaboration

Women are not referred back

Prevention of over-medicalization as key task

Bearing the weight of medical responsibility

Implications of low reliability of detection methods

Confidence in own capability and resilience

Discrepancy between recommendations and practical reality

**Maintaining professional confidence**

**Coping with a lack of trust in collaboration with secondary care**

**Adapting to the client care needs**

**Managing screening and detection uncertainty**

Variation and ambiguity of the guidelines and protocols

Struggling with FGR detection despite its key task

Validity of the screening methods

Deciding on third trimester screening strategy

Pregnant women need for reassurance about the baby’s health

Thoughtful communication with women is key

The hospital ultrasound is possibly of low quality

Coping with suboptimal collaboration

Accepting some women prefer secondary care

Societal shift to technology- and control

(Lack of) previous experiences

Fear of a perinatal audit and litigation

Being judged in hindsight

Population-specific differences in the desire for ultrasounds

Dealing with requests for ultrasounds

Figure S2 Code tree
